# Deep plasma proteomics identifies and validates an eight-protein biomarker panel that separate benign from malignant tumors in ovarian cancer

**DOI:** 10.1038/s43856-025-00945-0

**Published:** 2025-06-12

**Authors:** Mikaela Moskov, Julia Hedlund Lindberg, Maria Lycke, Emma Ivansson, Ulf Gyllensten, Karin Sundfeldt, Karin Stålberg, Stefan Enroth

**Affiliations:** 1 Department of Immunology, Genetics, and Pathology, Biomedical Center, SciLifeLab Uppsala, Uppsala University, Uppsala, Sweden; 2 Department of Obstetrics and Gynaecology, Institute of Clinical Sciences, Sahlgrenska Academy at Gothenburg University, Gothenburg, Sweden; 3 Department of Women’s and Children’s Health, Uppsala University, Uppsala, Sweden

**Keywords:** Diagnostic markers, Ovarian cancer

## Abstract

**Background:**

Ovarian cancer has the highest mortality of all gynecological cancers and surgery is commonly used as final diagnostic. Available literature indicates that women with benign tumors could often be conservatively managed, but accurate molecular tests are needed for triaging when gold-standard imaging techniques are inconclusive or lacking.

**Methods:**

Here, we analyzed 5416 plasma proteins in two independent cohorts (N_1_ = 171, N_2_ = 233) with women surgically diagnosed with benign or malignant tumors. Using one cohort as discovery, we compared protein levels of benign tumors with early stage (I–II), late stage (III–IV) or any stage (I–IV) ovarian cancer and trained risk-score reporting multivariate models including a fixed cut-off for malignancy. Associations and model performance was then evaluated in the replication cohort.

**Results:**

We identify 327 biomarker associations, corresponding to 191 unique proteins, and replicate 326 (99.7%). By comparing the 191 proteins with their corresponding tumor gene expression we find that only 11% (21/191) have significant correlation. Through analyzes of protein-protein correlation networks, we find that 62 of the 191 proteins have high correlation with at least one other protein, suggesting that many of the associations are secondary effects. In the replication cohort, our model has areas under the curve (AUC = 0.96) corresponding to 97% sensitivity at 68% specificity. For early-stage tumors, we estimate the sensitivity to 91% at a specificity of 68% as compared to 85% and 54% for CA-125 alone.

**Conclusions:**

Our results indicates that up to one third of benign cases can be identified by molecular measures thereby reducing the need for diagnostic surgery.

## Plain language summary

Ovarian cancer is the deadliest of all gynecological cancers, and surgery is commonly needed to get a confident diagnosis. The majority of women with symptoms have non-cancerous (benign) conditions that could be more conservatively managed. If available, accurate molecular tests could tell cancer from non-cancer without surgery. Here, we have looked at over 5400 blood protein markers in two Swedish cohorts. We detected and validated many markers in symptomatic women and found that a test based on eight biomarkers showed excellent performance in separating benign from malignant tumors regardless of tumor type and could have clinical use when classifying symptomatic women.

## Abstract (web summary)

Moskov et al. explored over 5400 blood protein biomarkers in relation to separating malign from benign ovarian tumours in two cohorts of symptomatic women. The study detected and validated many individual markers and developed a test based on eight biomarkers that showed excellent performance in classifying patients regardless of tumor type.

## Introduction

Ovarian cancer is the deadliest of all gynecological cancers and the 8th most common female cancer overall, with over 300,000 new cases and 200,000 deaths per year^1^. Discovery is mainly symptom-driven or by incidental finding^2^, and there are no biomarkers available today that could justify general screening^3^. The common late-stage diagnosis leads to an overall 5-year survival of ovarian cancer of only 30–50%. However, if the cancer is detected in stage I, close to 90% of patients can be cured, while patients with spread cancers detected in stage III or IV has a 5-year survival rate of less than 30%^4^. Few molecular biomarkers are clinically used today to complement imaging examinations, but none have sufficient accuracy to be used for screening or for accurate diagnostics in symptomatic women. Recent investigations based on single-cell mRNA sequencing have shown patient-specific gene expression patterns, specific changes in both gene-expression in the surrounding tumor micro-environment, as well as cell-type composition in relation to tumor progression^5^. The plasma proteome could potentially be a reflection of such protein expression changes in any of the affected cell types. Differentially expressed circulating protein biomarkers as detected in plasma could therefore stem from the actual tumor cells as well as from a larger variety of surrounding cell types directly or indirectly affected by the progressing tumor^6^.

When diagnostic imaging indicates adnexal mass, surgery is often necessary for a final diagnosis. Most of the adnexal mass cases are of benign nature^7,8^ and there are indications that these could be conservatively managed, e.g., without surgical intervention, or by choosing less invasive surgical procedures with low risk of complications^2^. Surgical intervention, by itself, is not risk-free and surgery-related complications have been reported in 3.5% to as high as 15% of the women with benign adnexal mass^2,9^. Imaging techniques can achieve high accuracy in separating benign from malignant conditions^10^, but as noted in a recent Cochrane review^11^, the bulk of the present literature evaluating the imaging techniques is based on studies conducted in tertiary settings, and the clinical setting has both a significant impact on the performance and the cost-benefit for the health care system. Molecular preoperative tools that accurately separate benign from malignant cases could help in reducing referrals to tertiary centers and unnecessary surgical interventions, thereby also minimizing potential complications and side effects, such as infertility or premature menopause^12^. Mucin-16 (CA-125 or MUC16) is currently the best single molecular biomarker used for ovarian cancer diagnosis in post-menopausal women and in treatment management^13^. MUC16, however, has low sensitivity for early-stage cancer and can also be elevated in many benign gynecological conditions in younger women, such as infections, pregnancies, and endometriosis^13,14^, resulting in a high proportion of false positives when discriminating between benign and malignant ovarian cancer tumors. MUC16 levels have also been found to be above the clinically indicative cut-off for ovarian cancer (35 U/mL) in close to half of women with acute pancreatitis^15^ and in one of 20 elderly women with heart failures^16^. Combining MUC16 with other biomarkers, including WAP Four-Disulfide Core Domain 2 (WFDC2 or HE4), as in the ROMA Score (Ovarian Malignancy Risk Algorithm) improves the performance. The ROMA score is calculated differently in pre- and post-menopausal women and has been reported with an overall sensitivity of 87.0% at a specificity of 80.9% in pre-menopausal and with 91.1 and 77.2 % in post-menopausal women respectively^8^. In early stages, the sensitivities in pre-/post-menopausal women have been reported at 77.8/81.4% at specificities of 80.9/77.2%^8^. The lower sensitivity at the indicated cut-off for detection of early-stage ovarian cancer (stages I and II) prohibits accurate discrimination of benign and malignant conditions in symptomatic women. Several studies have indicated that combining several protein biomarkers into a single test can increase test performance. The OVA1-test, for instance, combines five proteins (apolipoprotein A1, beta-2 microglobulin, MUC16, Transferrin and Prealbumin/Transthyretin) and classifies women into categories of high, intermediate or low risk of ovarian cancer. In a multi-center study^17^ a higher proportion of the individuals predicted to be low risk, e.g., having benign tumors, according to the OVA1-test remained benign during a 12-month follow-up period as compared to using MUC16 alone. A second generation of the OVA1-test, OVERA^18^, also combining five proteins (MUC16, transferrin, apolipoprotein A1, follicle-stimulating hormone, and WFDC2) increased the sensitivity to an estimated 69% at a specificity of 91%. OVERA is reported to have a sensitivity of 88.6% in detecting early-stage cancers (stages I and II)^18^. We have previously developed^19^ and validated^20^ an 11-protein biomarker panel that outperformed MUC16 in separating benign from malignant conditions at time of diagnose. This panel was constructed based on analyses of up to 983 plasma proteins and achieved sensitivities of 83–88% at specificities of 88–92% at a pre-defined cut-off across two independent validation cohorts with both pre- and post-menopausal women^19^. Additional studies of up to 1536 plasma proteins^21,22^ and up to 3072 proteins^23^ have identified multiple additional biomarker candidates for ovarian cancer with promising results, but additional validation is needed before clinical use. These studies indicate that combinations of biomarkers, even without inclusion of MUC16, can achieve high precision in separating benign from malignant conditions, and that large-scale characterizations of the plasma proteome in combination with machine learning represent a promising route for the development of novel tests for the separation of benign and malignant ovarian tumors. In this study, we aimed to identify and validate single and multiplex biomarkers for the separation of malignant and benign ovarian cancer tumors in symptomatic women. To this end, over 5400 plasma proteins in each sample were characterized using high-throughput affinity-based proteomics and RNA-sequencing in the corresponding tumor tissue was used to assess tumor gene-activity in relation to the plasma proteins. Finally, we employed machine learning to identify a small biomarker signature consisting of eight proteins that predicts malignancy, and the performance of this signature was validated in a separate cohort.

## Methods

### Study design

This study included clinical samples collected from two geographically separated locations in Sweden. Both cohorts consisted of women that have been surgically diagnosed with either benign or malignant tumors after suspicion of ovarian cancer. Samples were collected according to standardized protocols and according to local regulations. All participants gave written consent, and all necessary ethical permits were in place. One cohort was used strictly as a discovery and the second as a validation cohort. A high-throughput proteome assay was used to characterize the plasma proteome in both cohorts at the same time in the same laboratory. RNA-sequencing was used to characterize gene expression in fresh-frozen tumor tissue from a subset of the women in the validation cohort. Protein biomarkers were analyzed both individually and in combination in relation to clinical endpoints. Strict adjustment for multiple hypothesis testing was used throughout.

### Samples

Plasma samples of women with benign and malignant ovarian tumors were collected from the tumor biobank^24^ at Biobankvast.se, Western healthcare region, Göteborg, Sweden (*N* = 171) and from the *U*-CAN collection^25^ at Uppsala Biobank, Uppsala University, Sweden (*N* = 233). Inclusion criteria was suspicion of ovarian cancer followed by surgical diagnosis of either malignant or benign conditions. Exclusion criteria were patients that had received neoadjuvant treatment prior to surgery or if the tumor was pathologically determined to be metastases originating from other tissues. The samples from Göteborg were collected from 2016 to 2018 and the samples from *U*-CAN in Uppsala between 2012 and 2018. All samples were collected in agreement with local guidelines and regulations. The tumors were examined by pathologist specialized in gynecologic cancers for histology, grade, and stage according to International Federation of Gynecology and Obstetrics standards. Both cohorts contained mixed tumor histology. In the Göteborg samples, 74.1% were high-grade serous (HGSC), 9.4% low-grade serous (LGSC), 8.2% mucinous, and the remainder clear cell, endometroid, sarcoma, epithelial/clear cell or mucinous/teratoma. In the *U*-CAN samples, 66.5% were HGSC, 9.2% LGSC, 6.6% endometroid, 6.6% clear cell, 5.9% carcinosarcoma and the remainder mucinous, non-epithelial, endometroid, mixed and/or unclear. All plasma samples were frozen and stored at −70 °C. Basic statistics for the samples used are presented in Table 1 with additional cohort characteristics in Supplementary Data 1. The study was approved by the Regional Ethics Committee in Uppsala (Dnr: 2016/145) and Göteborg (Dnr: 201-15) and informed written consent was obtained from all participants following the guidelines of the Declaration of Helsinki.

**Table 1:** Cohort characteristics

	Cohort	All	Benign	Ovarian Cancer			
				I	II	III	IV
Nr. Samples	Discovery	171	86	13	11	38	23
	Replication	233	81	13	10	80	49
Age at Diag.^a^	Discovery	60.3 (14.6)	58.1 (16.6)	62.2 (11.5)	67.7 (8.7)	60.3 (12.4)	63.8 (12.4)
	Replication	61.5 (12.0)	58.2 (14.0)	61.7 (10.5)	67.6 (8.7)	62.0 (10.6)	64.8 (10.3)
Age diff pval^b^		0.71	0.79	0.96	0.97	0.76	0.74
Clin. CA-125^c^	Discovery	820.4 (1989.4)	85.8 (181.8)	107.3 (156.7)	546.3 (642.4)	1957.0 (3017)	2214.8 (2874.3)
	Replication	1175.3 (2356)	93.1 (138.4)	587.7 (862.9)	1006.8 (1122.6)	1354.0 (2610.3)	2309.9 (3176.2)
CA-125 diff pval^b^		0.71	0.79	0.96	0.97	0.76	0.74

^a^ Reported as mean (std dev) age in the group. ^b^ Two-sided Wilcoxon ranked test comparing the Discovery and Replication cohorts. ^c^ Clinically measured CA-125 at time of diagnosis, reported as mean (std dev) U/ml in the group.

### Plasma protein characterization

The plasma proteome was analyzed using the proximity extension assay (PEA) as implemented in the Explore HT-version (Olink Proteomics AB, Uppsala, Sweden). The samples were analyzed at the Olink Service Laboratory in Uppsala, Sweden. In brief, the PEA is based on pairs of antibodies equipped with probes, DNA single-strand oligonucleotide reporter molecules, that bind to their respective target if present. Target binding by both probes in close proximity generates double-stranded DNA amplicons, which are then quantified by next-generation sequencing^26^. Here, 5416 unique proteins were characterized in each of the 404 samples. The samples were randomized across plates with respect to cohort and diagnose (benign or malignant). In the resulting data file provided by the analysis platform, each individual protein measurement, assay and sample is labeled depending on passing or failing quality control as provided by the instrument software. Here, a total of 768 individual measurements (corresponding to 0.036%, 768/(404*5416)) did not pass quality control and were removed from further analyses. Two proteins (apolipoprotein E and fibronektin 1) had detection rates below 95% across all samples and were removed from further analyses. The resulting NPX values are on a log2 scale and in the logarithmic phase of the curve, one (1) increase of the NPX value corresponds to a doubling of the protein content. In the resulting data, a high NPX value corresponds to a high protein concentration. Each of the measured proteins has a lower limit of detection (LOD) given in the same NPX-scale, which is determined at run time. Here, measurements under LOD were kept as is in the downstream analysis. After quality control, the detection rate across all samples was 99.5 to 99.8%.

### Tumor RNA extraction

Fresh frozen tumor tissue samples from women in the Uppsala cohort was used for analysis of mRNA expression. Nucleic acids were isolated using the AllPrep® DNA/RNA Micro Kit (Cat.no. 80284, Qiagen, Hilden, Germany). From the fresh frozen tumor samples, approximately 2–5 cryosections (thickness 10 µm) were obtained, using a CryoStar NX70 Cryostat (Thermo Fisher Scientific™ Inc.). Cryosections were immediately transferred into 1,5 ml Eppendorf tubes containing 600 eμl Buffer RLT with addition of 1% β-mercaptoethanol (Cat.no. M6250-10ML, Merck, Darmstadt, Germany). Samples were homogenized by continuous vortexing for 30 s, followed by simultaneous extraction of total RNA and genomic DNA from each sample, according to the manufacturers protocol for microdissected cryosections. All RNA samples were subjected to DNase treatment using the RNase-Free DNase Set (Cat.no. 79254, Qiagen, Hilden, Germany), and finally eluted in RNase free water before storage at −80 °C. RNA yield and integrity were measured on the Agilent 2100 system, using the RNA 6000 Nano Kit (Cat.no. 5067–1511, Agilent Technologies, Santa Clara, CA, USA).

### mRNA sequencing and alignment

Sequencing libraries were prepared from 122–194 ng (three samples), 200 ng (seven samples) or 500 ng (71 samples) total RNA using the TruSeq stranded mRNA library preparation kit (cat# 20020595, Illumina Inc.) including polyA-selection. Unique dual indexes (cat# 20022371, Illumina Inc.) were used. The library preparation was performed according to the manufacturers’ protocol (#1000000040498, Illumina Inc.). The libraries were then sequenced on a NovaSeq 6000 system (Illumina Inc.) on S4 flowcells with version 1.5 sequencing chemistry on three lanes. Paired-end sequencing with read lengths of 150 bp was used. Across all sequenced samples, 112 to 477 M raw reads were obtained. The data was analyzed with the nf-core framework^27^ version 3.3 (https://nf-co.re/rnaseq/3.3). In brief, the reads were aligned to the human reference genome (GRCh38) using the STAR software suite and 82 to 221 M aligned reads were obtained for each sample. The Ensembl database was used for annotation of genes and the transcript per million (TPM) value was used as representation of gene expression.

### Statistics and reproducibility

All analyses were carried out in R (version 4.2.3)^28^. The univariate comparisons were done one protein at a time using a two-sided Wilcoxon ranked based test. The resulting *p* values were adjusted for multiple hypothesis testing using the Holm correction method as implemented in the ‘‘p.adjust’’ *R*-function. The correlations between each plasma protein and corresponding tumor gene expression were calculated using the ‘‘cor.test’’ function in R with the Spearman method and resulting *p* values adjusted using the Holm method as above. The protein-protein correlations were calculated using the Spearman method via the ‘‘cor.test’’ function in *R*. This analysis was restricted to protein pairs in which at least one protein was among the 191 univariate significant proteins, and the *p* values were adjusted using Holm’s method as above. Using proteins that had significant (*q* < 0.05) absolute correlations of at least 0.8 in the discovery cohort, the network and clusters were built using the ‘‘igraph’’ R package^29^ with clusters identified using the Leiden method based on modularity. In the visualization, network nodes were scaled between one and 20, and the clusters between three and 50, by the number of degrees. Prior to the multivariate analysis, NPX-values were normalized between cohorts by first calculating a per-protein normalization factor as the difference of the mean of the benign between the discovery and the replication cohort. This normalization factor was then added to each individual measurement in the replication cohort. Multivariate models were built using the discovery cohort only. Three separate models were created: early-stages (I and II), late-stages (III and IV) and any stage ovarian cancer (I–IV) versus benign, respectively, in the same way. Starting from the 191 univariately significant proteins, a feature selection step was first done by recursive feature elimination as implemented in the ‘‘rfe’’ function in the ‘‘caret’’ *R*-package^30^. Distance weighted discrimination^31^ with a polynomial kernel from the ‘‘kerndwd’’ (version 2.0.3) *R* package^32^ was then used to create a prediction model. A tuning step was performed during training over the ‘‘lambda’’, ‘‘qval’’, ‘‘degree’’, and ‘‘scale’’ parameter. The final three models used the following sets for these parameters early stage: (0.01, 0.05, 1, 0.1), late stage: (0.1, 0.05, 1, 0.1) and any stage: (0.1, 0.05, 3, 0.1). The output from the model was risk-score on the scale 0 to 1 and ROC-curves was generated using the ‘‘pROC’’ *R*-package^33^. A cut-off for malignancy was then developed using the ROC-curve from the discovery cohort and taken at the first point on the curve with at least 95% sensitivity. The model was then applied to the replication samples and a decision was made based on the cut-off from the discovery cohort. Obtained AUCs were compared between the discovery and replication cohort using the ‘‘roc.test’’ function from the ‘‘pROC’’ *R*-package^33^ with the DeLong’s method. Obtained sensitivities and specificities at the cut-off was compared between the discovery and replication cohorts using a Fisher’s exact test.

### Reporting summary

Further information on research design is available in the Nature Portfolio Reporting Summary linked to this article.

## Results

### Deep plasma proteome characterization

**Fig. 1:**
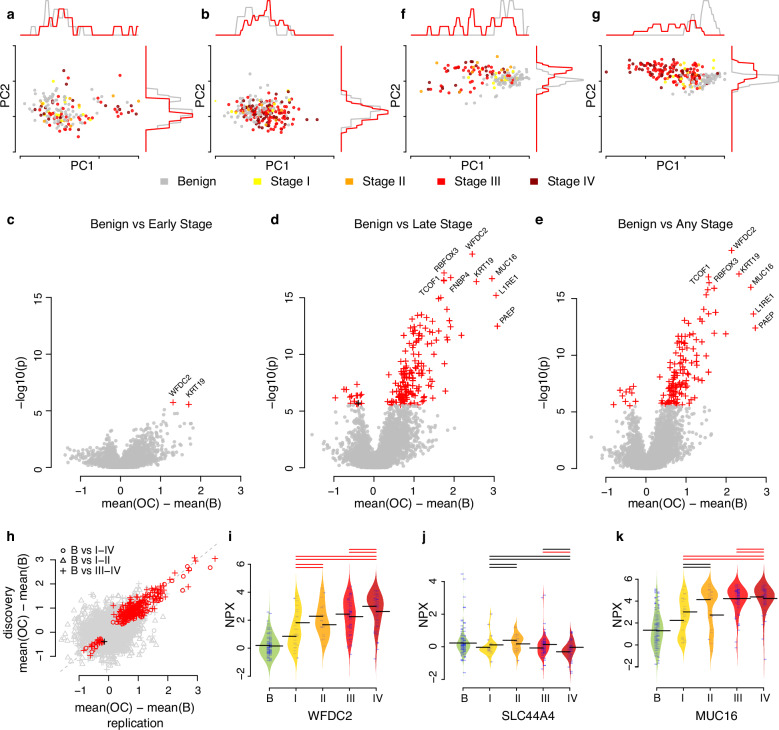
Protein biomarker candidates for ovarian cancer. **a** PCA-projection of the discovery cohort using all proteins. Individual samples are labeled based on diagnosis. **b** Projection (PCA) of the replication cohort using the same transformation in (**a**). **c** Volcano-plot showing mean difference between early-stage ovarian cancer and benign diagnoses on *x*-axis and statistical significance (two-sided Wilcoxon ranked sum test) on the *y*-axis in the discovery cohort. Proteins with significant difference (*q* value < 0.05, adjusted with Holm’s method) in the discovery cohort are drawn as crosses and colored by statistical significance in the replication cohort, with red indicating replicated and black not replicated. **d** as (**c**) but for benign diagnoses compared to late-stage ovarian cancer. **e** as (**c**) but for benign diagnoses compared to all stages of ovarian cancer. **f** as (**a**) but using significant biomarkers (two-sided Wilcoxon ranked sum test, *q* value < 0.05, adjusted with Holm’s method) only. **g** Projection (PCA) of the replication cohort using the same transformation in (**f**). **h** Comparisons of the fold changes between cases and controls in the discovery cohort (y-axis) and the replication cohort (*x*-axis). Proteins with significant difference (*q* value < 0.05, adjusted with Holm’s method) are drawn as crosses and colored by statistical significance also in the replication cohort with red indicating replicated and black not replicated. **i** WCFD2 protein abundance in plasma in relation to diagnose and stage (*x*-axis). Each ‘‘beanplot’’ show distribution of discovery cohort to the left and the replication cohort to the right. Thick black horizontal lines indicate mean in each group with solid lines for the discovery and dashed lines for the replication cohorts respectively. Thin lines above the beanplots indicate statistical significance between the spanned stages and the benign group with red indicating *q* value < 0.05 and black > 0.05. In each set of lines, the dashed line is for the replication cohort and the solid line is for the discovery cohort. **j** as (*i*) but for SLC44A4. **k** as (*i*) but for MUC16.

The plasma proteome of 404 women, surgically diagnosed with either benign or malignant conditions after suspicion of ovarian cancer, was studied, utilizing the PEA implemented in the Olink Explore HT assay^34^. The samples were collected from two independent Swedish cohorts (Table 1) at time of diagnose, before initiation of treatment. The samples were collected at two different geographical locations, with the samples collected in Göteborg used as discovery cohort and the samples collected in Uppsala as replication cohort. Both cohorts contained mixed tumor histology with HGSC as the most common with 74.1 and 66.5% incidence, respectively (Supplementary Data 1). A total of 5416 unique proteins was characterized in each sample. A flowchart illustrating our analysis pipeline can be seen in Supplementary Fig. 1. After basic quality control (see Methods) requiring at least 95% detection rates in both individuals and proteins, 5414 proteins in all 404 individuals were included in further analyses. Both cohorts have clinically measured CA-125 (Table 1), and a significant (*p* < 1.3 × 10–44) correlation was found between clinical CA-125 and the MUC16 assay on the Olink Explore HT, with an estimated correlation coefficient of 0.74 (Spearman’s Rho). The protein content on the assay reflects a broad spectrum of biological processes and functions, not only with previous relation to cancer, and in line with this, using all 5414 proteins we observed no clear distinction between benign and malignant diagnosis using the first two PCA dimensions (Fig. 1A) in the discovery cohort with a total of 15% of the total variance explained by the first two principal components. A similar pattern was seen when projecting the replication cohort onto the same plane (Fig. 1B).

### Replicated single protein biomarkers candidates for ovarian cancer

Using the discovery cohort, each of the 5414 proteins was compared in three different setups: benign vs early-stage ovarian cancer (stages I and II), benign vs late-stage ovarian cancer (stage III and IV), and benign vs any stage ovarian cancer (stages I, II, III, or IV). Across all 16242 comparisons (5414 proteins × 3 comparisons), we found 327 significant associations (*q* value < 0.05, adjusted for multiple hypothesis testing with the Holm’s method) in the discovery cohort (Supplementary Data 2). The 327 associations corresponded to a total of 191 unique proteins with two proteins (keratin-19 (KRT19) and WFDC2) found to be significantly different in all three comparisons (Fig. 1C–E). Each of the 327 associations was then examined using the replication cohort, and 326 (99.7%, Fig. 1C–E) remained significant after adjustment for multiple hypothesis testing (Holm’s method). The one protein that did not replicate, solute carrier family 44 member 4 (SLC44A4), showed a significant association only between late-stage cancer and benign tumors in the discovery cohort (Supplementary Data 2). Using all the 191 proteins that showed a significant difference in the discovery cohort, we observed a trend towards a distinction between benign and malignant diagnoses in the first two PCA dimensions in the discovery cohort with a total of 53% of the total variance explained by the first two principal components (Fig. 1F), as well in the replication cohort (Fig. 1G) when projected onto the same plane. We next investigated the fold-change between the benign and malignant groups for the 327 associations and found a high similarity (Fig. 1H, Pearson’s Rho 0.93, p < 3.0 ×10^−140^), both for biomarkers with a higher expression in those with malignant versus those with benign diagnoses and vice versa (Fig. 1H). We also observed different patterns among the protein biomarkers in relation to the cancer stage, with e.g., increasing levels with stage or non-linear plateauing. Figure 1I–K shows the observed distribution of protein levels in benign and malignant diagnoses for both the discovery and replication cohort for three proteins. Figure 1I shows the top ranking hit overall, WFDC2, with increasing levels for stages I-IV compared to benign. Figure 1J shows the only protein that did not replicate, SLC44A4 and Fig. 1K shows the patterns observed in MUC16. Note that MUC16 was not significantly different between benign and early cancer stages in the discovery cohort nor the replication cohort. All results for the three comparisons are reported in Supplementary Data 2. In conclusion, we found a large number of potential plasma protein biomarkers out of which over 99% validated in a replication cohort. Not all biomarkers however showed a difference across both early and late cancer stages as compared to benign tumors and few displayed a clear linear relationship with cancer stage.

### Plasma protein biomarker levels are in general not correlated with tumor gene expression

**Fig. 2:**
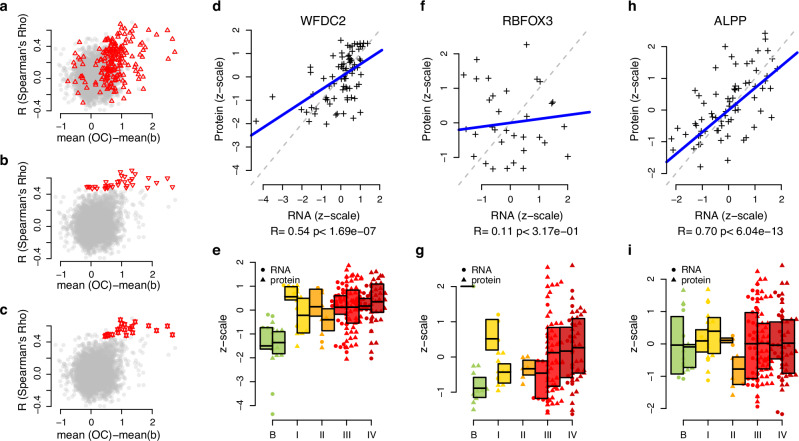
Plasma protein abundance and tissue gene expression. **a** Correlation (*y*-axis, Spearman’s Rho) between plasma protein abundance (NPX) and tissue gene expression (TPM) compared to mean difference between benign and malignant diagnoses (*x-*axis) for all proteins. Red triangles indicate the significant (two-sided Wilcoxon ranked sum test, *q* value < 0.05, adjusted with Holm’s method) difference in the discovery cohort between benign and malignant diagnoses. **b** As (**a**) but red triangles indicate significant correlation (two-sided Spearman test, *q* value < 0.05, adjusted with Holm’s method) between plasma proteins and tissue gene expression. (**c**) as (B) but red double triangles are overlapping features from (**a**) and (**b**). **d** Protein plasma abundance levels (*y*-axis) and tissue RNA expression levels (log2, *x*-axis) for WFDC2. Both axes are *z*-scaled to have mean = 0 and unit variance. Labels below the *x*-axis is the Rho estimate and the corresponding *p* value (two-sided Spearman test). The thick blue line represents the Spearman’s correlation coefficient. **e** Boxplots with individual protein (right) and RNA abundance (left) levels for benign (labeled ‘‘B‘‘) and malignant (stages I–IV) diagnoses for WFDC2 The top and the bottom of the box represent the 25 and 75th percentile and the band inside the box the median value. (**f**) and (**g**) as (**d**) and (**e**) but for RBFOX3. (**H**) and **(i)** as (**d**) and (**e**) but for ALPP.

The tumor microenvironment is a complex environment and circulating protein biomarkers could stem from the actual tumor cells or from a larger variety of surrounding cell types directly or indirectly affected by the progressing tumor^6^. To assess if the plasma protein levels reflect the gene expression pattern in the tumor, we characterized the gene-expression in 81 tumor samples from the replication cohort with total RNA-sequencing (see Methods). This included samples of both benign (*N* = 10) and malignant tumors (*N* = 71, *N* stages I–IV: 5, 5, 37, 24). Using the TPM score we then calculated the correlation coefficient between tumor mRNA expression and the plasma protein levels. We found non-zero gene expression levels in at least one sample for 5364 of the 5414 proteins (99.1%). When comparing the corresponding tumor gene expression with the plasma protein levels, 33 protein-gene pairs, corresponding to 0.62% (33/5364, Table 2), were found to have a significant correlation in a paired analysis (Spearman’s Rho, multiple hypothesis adjusted *q* value < 0.05, Supplementary Data 3). When examining the 191 proteins whose levels were found to be significantly different between benign and malignant tumors, 21 proteins (Table 2), corresponding to 11.0% (21/191), showed a significant correlation with the corresponding tumor mRNA expression. This represents a significantly higher proportion than in the comparison of all proteins examined (*p* < 6.8 × 10^−20^, binominal test). Across the 191 proteins, both proteins with an increase or a decrease in plasma protein concentration in malignant tumors as compared to benign were found to have both positive or negative correlations with their corresponding tumor gene-expression (Fig. 2A). Among the 33 proteins showing a significant correlation between plasma and RNA levels, however, only positive correlations were observed (Fig. 2B). When requiring both significant difference in plasma proteins expression between malignant and benign diagnoses and significant correlation with tumors mRNA expression, only positively correlated pairs with higher plasma protein concentrations in malignant compared to benign diagnoses was observed (Fig. 2C). Among the top-ranked plasma protein biomarkers in the univariate analysis we found examples of both correlated and non-correlated patterns. For instance, WFDC2 plasma levels were found to be significantly correlated (*p* < 1.7 × 10^−7^) with corresponding mRNA expression in the tumor (Fig. 2D, E). On the other hand, the plasma level of the RNA binding fox-1 homolog 3 (RBFOX3) was not correlated (*p* = 0.32) with tumor mRNA expression (Fig. 2F, G). Alkaline phosphatase, placental type (ALPP) showed the strongest correlation between protein level and tumor mRNA expression (Spearman’s Rho = 0.70, *p* value < 6.0 × 10^−13^, Fig. 2H). ALPP did not, however, show any difference in protein expression between malignant and benign diagnoses (Fig. 2I). These results indicate that correlation between tumor gene expression and plasma protein level is neither a necessity nor an assurance for a strong univariate plasma protein biomarker for ovarian cancer.

**Table 2:** Plasma proteins with significantly correlated tumor RNA expression

Protein	Gene name	Gene ID^a^	R^b^	*p* value^c^	Plasma Biomarker^d^
Alkaline phosphatase, placental type	*ALPP*	ENSG00000163283	0.695	6.0 × 10^−13^	No
Kallikrein-6	*KLK6*	ENSG00000167755	0.676	4.5× 10^−12^	Yes
Folate receptor alpha	*FOLR1*	ENSG00000110195	0.650	5.0 ×10^−11^	Yes
Matrilysin	*MMP7*	ENSG00000137673	0.629	3.1 ×10^−10^	Yes
Kallikrein-10	*KLK10*	ENSG00000129451	0.618	8.1 ×10^−10^	Yes
V-set domain-containing T-cell activation inhibitor 1	*VTCN1*	ENSG00000134258	0.601	2.9 × 10^−9^	Yes
Kinesin-like protein KIFC1	*KIFC1*	ENSG00000237649	0.598	3.9 × 10^−9^	Yes
Keratin, type I cytoskeletal 18	*KRT18*	ENSG00000111057	0.591	6.2 × 10^−9^	Yes
Ribonucleoside-diphosphate reductase subunit M2	*RRM2*	ENSG00000171848	0.568	3.2 × 10^−8^	Yes
Kunitz-type protease inhibitor 1	*SPINT1*	ENSG00000166145	0.563	4.4 × 10^−8^	Yes
Mesothelin	*MSLN*	ENSG00000102854	0.554	8.2 × 10^−8^	Yes
Glutathione S-transferase theta-2B	*GSTT2B*	ENSG00000133433	0.552	9.1 × 10^−8^	No
Citron Rho-interacting kinase	*CIT*	ENSG00000122966	0.552	9.5 × 10^−8^	Yes
WAP four-disulfide core domain protein 2	*WFDC2*	ENSG00000101443	0.542	1.7 × 10^−7^	Yes
Nucleus accumbens-associated protein 1	*NACC1*	ENSG00000160877	0.516	8.1 × 10^−7^	Yes
Treacle protein	*TCOF1*	ENSG00000070814	0.515	8.8 × 10^−7^	Yes
Odorant-binding protein 2b	*OBP2B*	ENSG00000171102	0.513	9.9 × 10^−7^	No
G1/S-specific cyclin-E1	*CCNE1*	ENSG00000105173	0.504	1.6 × 10^−6^	No
Alkaline phosphatase, germ cell type	*ALPG*	ENSG00000163286	0.502	1.8 × 10^−6^	No
Basal cell adhesion molecule	*BCAM*	ENSG00000187244	0.496	2.5 × 10^−6^	Yes
Polymeric immunoglobulin receptor	*PIGR*	ENSG00000162896	0.495	2.7 × 10^−6^	No
Kallikrein-7	*KLK7*	ENSG00000169035	0.494	2.8 × 10^−6^	No
Collagen alpha-1(XXIV) chain	*COL24A1*	ENSG00000171502	0.491	3.2 × 10^−6^	Yes
Disintegrin and metalloproteinase domain-containing protein 23	*ADAM23*	ENSG00000114948	0.490	3.4 × 10^−6^	No
Clusterin-like protein 1	*CLUL1*	ENSG00000079101	0.489	3.6 × 10^−6^	No
Glycodelin	*PAEP*	ENSG00000122133	0.489	3.6 × 10^−6^	Yes
Kallikrein-8	*KLK8*	ENSG00000129455	0.484	4.7 × 10^−6^	Yes
Mitochondrial proton/calcium exchanger protein	*LETM1*	ENSG00000168924	0.482	5.2 × 10 ^−6^	Yes
Melanoma-associated antigen 4	*MAGEA4*	ENSG00000147381	0.480	5.8 × 10^−6^	No
Cadherin-6	*CDH6*	ENSG00000113361	0.478	6.3 × 10^−6^	Yes
Stromelysin-2	*MMP10*	ENSG00000166670	0.471	9.0 × 10^−6^	No
Cadherin-3	*CDH3*	ENSG00000062038	0.471	9.2 × 10^−6^	No
G-patch domain and KOW motifs-containing protein	*GPKOW*	ENSG00000068394	0.471	9.3 × 10^−6^	Yes

^a^ Ensemble gene-id release 110. ^b^ Spearman’s Rho. ^c^ Two-sided, Spearman, raw. ^d^ Among the 191 protein plasma biomarker candidates.

### Correlation of plasma proteome reveals clusters of co-expressed networks

**Fig. 3:**
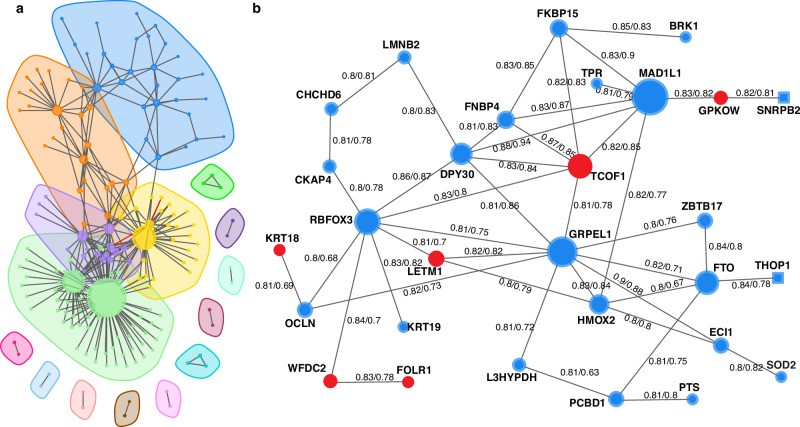
Correlation networks of plasma proteins. **a** Clusters of correlated protein-protein pairs as identified in the discovery cohort. Each node represents a unique protein with edges representing correlated protein pairs. Circular nodes correspond to proteins with significant difference between benign and malignant diagnoses in the discovery cohort (two-sided Wilcoxon ranked sum test, *q* value < 0.05, adjusted with Holm’s method) while nodes drawn as squares represent non-significant differences. Red edges represent correlations significant only in the discovery cohort. Shaded areas represent generated clusters based on the Leiden algorithm (Methods). **b** Detailed representation of one identified cluster from (**a**). Protein identifiers are written out next to each node and numbers on the edges represent correlation coefficients (Spearman’s Rho) in the discovery cohort and the replication cohort. As in (**a**), node shape corresponds to statistical significance in case control comparisons. Nodes are colored by RNA-protein correlation where red nodes represent plasma proteins with significant correlation to the corresponding tumor gene expression.

We next investigated protein-protein correlations in relation to ovarian cancer. Starting from the 191 proteins, we calculated protein-protein correlations in relation to all 5414 measured proteins using both the malignant and benign cases of the discovery cohort. We found a total of 106,101 significant protein-protein correlations (*q* value < 0.05, adjusted for multiple hypothesis testing with Holm’s method) in the discovery data out of which 95.8% (101,671) were found to be nominally significant (*p* value < 0.05) also in the replication data (Supplementary Data 4). After adjustment for multiple hypothesis testing also in the replication cohort (Holm’s method), 70.9% (75,259) remained significant (*q* value < 0.05). Based on the significant correlations with an estimated coefficient (Spearmans’ Rho) greater than 0.8, or smaller than −0.8, in the discovery cohort, we then built correlation networks. The highest estimated correlation factor found for MUC16 was 0.72 (with carboxypeptidase A4 (CPA4), Supplementary Data 4) and MUC16 was therefore not included in the network analysis. The network analysis resulted in 177 individual proteins connected by 315 strong correlations, out of which 62 proteins were among the 191 univariate significant biomarker candidates. In the network analysis, only two of the 315 correlations were not significant also in the replication cohort (frataxin to proteasome activator complex subunit 2 (PSME2) and cilia and flagella associated protein 36 (CFAP36) to PSME2). From these general networks, clusters of correlated sub-networks were identified (Methods). This analysis resulted in 15 sub-clusters of interconnected proteins (Fig. 3A, Supplementary Data 5). Five of these 15 clusters contained more than three proteins, ranging from 17 to 59 proteins each. One of the clusters is shown in detail in Fig. 3B, incorporating 29 proteins out of which all but two (thimet oligopeptidase 1, THOP1 and small nuclear ribonucleoprotein polypeptide B2, SNRPB2) were among the 191 proteins found to be significantly different between benign and malignant tumors (Fig. 3B). This network also contained six proteins (WFDC2, treacle ribosome biogenesis factor 1 (TCOF1), folate receptor alpha (FOLR1), leucine zipper and EF-hand containing transmembrane protein 1 (LETM1), *G*-patch domain and KOW motifs (GPKOW), and keratin 18 (KRT18)) that showed a significant correlation between plasma protein levels and mRNA tumor expression (Fig. 3B, Table 2). These results show that, although individual proteins have a significant difference in expression between the malignant and benign groups, several proteins pairs amongst the univariate significant biomarkers are observed to be closely co-expressed. This in turn suggests that the difference between malignant and benign diagnoses could be explained by a subset of the candidates.

### High-accuracy multivariate risk-score separates benign from malignant tumors

**Fig. 4:**
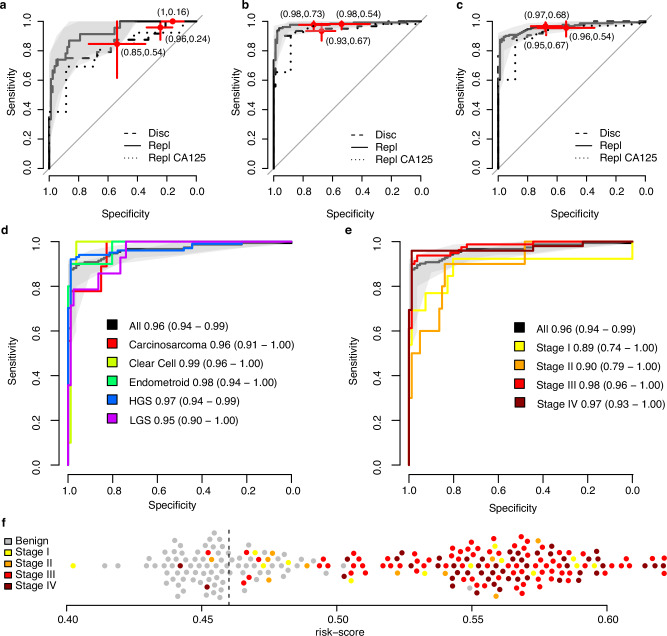
Multivariate prediction models. **a** Performance of the benign vs early stage (stages I and II) tumors prediction model. Receiver operating characteristic (ROC) curves are shown for the prediction model in the discovery cohort (dashed line) and in the replication cohort (solid line). The shaded area (gray) corresponds to the 95% confidence interval of the ROC curve in the replication cohort. The red crosses are centered on the point-estimate of the sensitivity and specificity obtained at the cut-off with the horizontal and vertical lines corresponding to the 95% confidence interval of the estimate. The panel also show the performance of clinically measured CA-125 (dotted line) in the replication cohort with the red cross as for the discovery and replication cohort but at a cut-off of 35 U/ml. **b** As (**a**) but for the benign vs late stage (stages III and IV) tumors. **c** As (**a**) but for the benign vs any stage (stages I–IV) tumors. **d** ROC curves for stratified analysis of different histology (*N*_HGSC_ = 101, *N*_LGSC_ = 14, *N*_Endometroid_ = 10, *N*_Clear cell_ = 10, *N*_Carcinosarcoma_ = 9) in the replication cohort for the benign vs any stage (stages I-IV) tumors. The solid black curve and shaded gray area as in (**c**), while the colored curves correspond to a subset of the samples split on histology. The label specifies the corresponding group and the point estimate of the AUC with the 95% confidence interval written out in parentheses. **e** As (**d**) but for a stratified analysis split on tumor stage (*N*_Stage I_ = 10, *N*_Stage II_ = 13, *N*_Stage III_ = 80, *N*_Stage IV_ = 49). **f** Individual risk-scores from the model in (**d**) for the replication cohort. The vertical dashed line indicates the cut-off used.

Following our analysis strategy above for the univariate comparisons we built three different multivariate models aiming at separating benign tumors from early stages (I and II), late stages (III and IV) and any stage (stages I–IV) ovarian cancer, respectively. As above, the Göteborg cohort was used as discovery cohort for model development (training) and for selection of a cut-off for predicting malignant condition. The models generate a risk-score on the scale of 0 to 1 and the cut-offs were chosen at a sensitivity of at least 95%. The models were created starting from the 191 proteins with univariate significance in the discovery cohort. As many of the proteins are correlated between themselves, we first used a supervised feature selection (see Methods) to limit the number of proteins in each model. The selected proteins were then used to build predictive models employing distance weighted discrimination^31^, a non-linear regression modeling framework for high-dimensional, low sample-size settings. A non-linear modeling framework was chosen based on the patterns observed in individual proteins in relation to the outcome as illustrated in e.g., Fig. 1I, K. The three models were trained and tuned using the discovery cohort (see Methods). The final model for separating benign vs early stage cancer was based on five proteins (keratin 19 (KRT19), FOLR1, WFDC2, BRICK1 subunit of SCAR/WAVE actin nucleating complex (BRK1), and V-set domain containing T-cell activation inhibitor 1 (VTCN1)), the final model for separating benign vs late stage cancer included seven proteins (WFDC2, MUC16, KRT19, TCOF1, crumbs cell polarity complex component 2 (CRB2), RBFOX3, and LINE1 retrotransposable element 1 (L1RE1)), and the final model for separating benign vs any stage cancer included eight proteins (WFDC2, KRT19, MUC16, RNA polymerase-associated protein LEO1 (LEO1), TCOF1, cysteine rich secretory protein 3 (CRISP3), FOLR1 and RBFOX3). In total, twelve proteins were included in any of the models, with only WFDC2 and KRT19 common to all three models (Table 3). We also calculated the relative importance of each protein in the separate multivariate models and found slight differences between the models although similar scores were obtained (Table 3). After training, the models were then evaluated in the replication cohort (Fig. 4A, B, C, Table 4). We found no statistical difference between the performances in the discovery and replication cohort for AUC (all *p* values > 0.21, Table 4) nor for the estimated sensitivities (all p-values 0.39, Table 3) or specificities (all *p* values > 0.25) at the respective cut-offs as indicated above. We also compared the performance of our models to that of clinically measured CA-125 in the replication cohort (Fig. Fig.4A–C). We could observe higher AUCs for all the multivariate models as compared to CA-125, but we found no statistical difference between the respective models and CA-125 (Fig. 4A, B, C, all *p* values > 0.14). The model separating any stage from benign conditions is the most applicable in clinical setting, before surgical diagnose and known tumor stage. Therefore, we evaluated the performance of that model when applied specifically to different subgroups in the replication cohort. First, we compared different groups with respect to histology and tumor stage (Fig. 4D, E). We found no statistical difference in the AUCs for the different histology subgroups as compared to the general performance (all *p* values > 0.14), with AUC ranging from 0.90 to 0.99 (Fig. 4D). A similar pattern was observed for the subgrouping of tumor stages compared to the general performance (all p-values > 0.24), with AUC ranging from 0.89 to 0.98 (Fig. 4E). Specifically, when applying the any stage model to early and late-stage samples separately in the replication cohort, sensitivities of 0.91 and 0.98, respectively, were observed (Table 4). Next, we compared the sensitivities and specificities obtained at the indicated cut-off in our model with the sensitivities and specificities obtained with CA-125 at the commonly used cut-off of 35 U/ml (Fig. 4F). The comparison revealed similar point estimates of sensitivities across all samples (model +1.1%) and for the late-stage group (model −0.51%) with a slightly higher point estimate for the early-stage group (model + 6.7%). Looking at the benign cases only, the point estimate of the specificity for the model (67.9%) was 14.1% higher than for CA-125 (53.8%). Using McNemar’s test for non-inferiority in paired samples we found no differences for the sensitivities (*p* = 1.0) nor for the specificities (*p* = 0.45) when comparing the predictions made by our model to CA-125. Finally, we analyzed the individual classifications of the any stage from benign model in the replication cohort. At the developed cut-off, the model correctly classified 67.9 % (55 of 81) of the benign samples, while miss-classifying 3.3% (five of 152) of the malignant tumors as benign (Fig. 4G). Among these five false negative samples, one sample each was from patients with a stage I, II and III tumor, respectively, and two samples were from patients with a stage IV tumor. Four of the five (80%) false negatives were classified as HGSC which is not statistically different (*p* value = 1, binomial test) from the proportion of HGSC in the whole replication cohort. The small number of false negatives prevented any additional stratified analysis of the sample group. In summary, our multivariate model displays robust performance in the replication across both tumor subtypes and stages with and estimated 14% higher specificity as compared to CA-125 alone.

**Table 3:** Proteins in multivariate models

	Variable Importance^a^		
Protein name (abbreviation)	Benign vs Stages I–II	Benign vs Stages III–IV	Benign vs Stages I–IV
WAP four-disulfide core domain protein 2 (WFDC2)	0.819	0.939	0.905
RNA binding protein fox-1 homolog 3 (RBFOX3)	-	0.918	0.872
Mucin-16 (MUC16)	-	0.911	0.867
Treacle protein (TCOF1)	-	0.910	0.878
Keratin, type I cytoskeletal 19 (KRT19**)**	0.814	0.908	0.881
LINE-1 retrotransposable element ORF1 protein (L1RE1**)**	-	0.892	-
Protein crumbs homolog 2 (CRB2**)**	-	0.864	-
RNA polymerase-associated protein LEO1 (LEO1**)**	-	-	0.843
Folate receptor alpha (FOLR1)	0.745	-	0.838
Protein BRICK1 (BRK1**)**	0.788	-	-
V-set domain-containing T-cell activation inhibitor 1 (VTCN1)	0.746	-	-
Cysteine-rich secretory protein 3 (CRISP3**)**	-	-	0.730

^a^A relative number on the scale of 0 to 1 ranking the individual importance of the proteins in each multivariate model. Rows in the table are sorted by the highest number across the three models.

**Table 4:** Predictive performance of the multivariate models

Model	Cohort	AUC	Sens^a^	Spec^a^
Early	Disc	0.85	0.96	0.24
	Repl	0.93	1.00	0.16
	D vs R pval^b^	0.21	1.00	0.25
Late	Disc	0.95	0.90	0.98
	Repl	0.98	0.91	0.98
	D vs R pval^b^	0.23	0.39	0.50
Any	Disc	0.96	0.95	0.67
	Repl	0.96	0.97	0.68
	D vs R pval^b^	0.79	0.52	0.43
	Repl (Early)^c^	0.89	0.91	0.68
	Repl (Late)^c^	0.97	0.98	0.68

^a^ Sensitivity (Sens) and Specificity (Spec) achieved at a cut-off defined using the discovery cohort requiring at least 0.95 sensitivity. ^b^ P-values calculated with the DeLong’s method for AUCs and Fisher’s Exact test for Sens and Spec. ^c^ Point estimates of the performance of the ‘Any stage’ model when applied to Early- or Late-stage samples in the replication cohort.

## Discussion

Detection of ovarian cancer is largely symptom driven, resulting in a considerable fraction of the cancer being discovered in late stages, which in turn leads to low 5-year survival rates. Far from all examined women, however, are diagnosed with malignant tumors. Even at secondary centers in Sweden, when imaging techniques indicate adnexal ovarian mass, up to 75% of symptomatic women are surgically diagnosed with benign conditions^8^. The surgery itself is not risk-free and apart from complications related directly to the operations there are additional considerations regarding effects on fertility and induced menopause^12^. Further, if there is a strong indication of ovarian malignancy, the patient should be referred for surgery at a university hospital. Molecular tests that could complement imaging techniques for separating benign from malignant conditions with high accuracy would benefit the patients themselves and provide an opportunity for health-care systems to reduce the workload for the highly trained experts, primarily at tertiary centers, that interpret, e.g., transvaginal ultrasounds. It has though, been difficult to find accurate enough biomarkers for ovarian cancer both with respect to screening and for triaging of symptomatic women.

In this study, we employed high-throughput ultra-sensitive affinity-based proteomics to perform unbiased searches for both univariate and multivariate biomarkers that could potentially separate malignant ovarian tumors from benign ones in symptomatic women. We found large differences in the plasma proteome between the benign and malignant groups and these effects were replicated in an independent cohort. We also concluded that only a small proportion of these protein biomarkers correlated with gene-expression in corresponding tumor tissue suggesting that the observable effects are possibly due to processes distinct from the developing tumor itself. This is in line with contemporary analyses of single-cell RNA expression highlighting the role of the tumor microenvironment in relation to tumor progression^6^. In the sense of liquid biopsy-based biomarkers, however, the ability to distinguish between diagnoses surpasses the need to know the exact origin of the protein. Indeed, here, only three out of the eight proteins in our multivariate signature displayed significant correlation with their respective mRNA expression in tumor tissue. In addition to replicating the performance of this multivariate signature in unseen samples with the AUC, we also validated the specific performance as indicated by sensitivity and specificity at a pre-determined cut-off level.

Our previously validated^20^ multiplex protein biomarker signature was developed for the same clinical application as here, but with a much smaller number of available assays, 983 compared to the 5416 measured here. The previously developed signature combined 11 proteins out of which four (WFDC2, KRT19, MUC16, FOLR1) overlap with the presented 8-protein signature. The remaining seven proteins were also measured with the assay used here, but the machine learning approach prioritized other proteins over these. Comparing the performances of these two signatures is not completely straight forward, partly because of technical differences in the assay but primarily on how the cut-offs were developed. In terms of AUCs, our previous 11-panel achieved AUCs of 0.95 and 0.92 in the development and validation cohorts respectively, while our 8-protein panel achieved AUCs of 0.96 in both cohorts. We previously used three cut-offs depending on intended use, one which focused on sensitivity, one for specificity and finally one providing the best balance between the two. Here, we focused on one type, requiring at least 95% sensitivity. At this cut-off, the performance in the replication cohort was estimated at a sensitivity of 97% with a specificity of 68%. Our 11-protein model was reported^20^ to have a sensitivity of 97% at a specificity of 20% in the validation phase, calculated at the cut-off which was focused on sensitivity. It should be noted that this focused cut-off was developed for a target at 98% sensitivity, so the comparison is not completely fair. Based on analyses of 3072 proteins we recently reported^23^ on a 3-protein model targeting the same clinical question as here. Since parts of the replication cohorts overlap between that study and the current, a direct comparison of the two models can be done. In the overlapping samples, the 8-protein model achieved and AUC of 0.97 while the 3-protein model had an AUC of 0.93. Overall, this different was not significantly different (*p* = 0.15, DeLong’s test, Supplementary Fig. 2A). Importantly however, the 8-protein model was found to have a significantly higher specificity (0.086 vs 0.058, *p* = 0.025, DeLong’s test) in the partial AUC range of 0.9 to 1.0 sensitivity (Supplementary Fig. 2B). In addition to an estimated higher performance in these measures, a major benefit of the models presented here is the low number of proteins which facilitates a clinical implementation, both in terms of cost but also in terms of controlling variance and repeatability in the assay itself. In practice, several additional challenges apply when characterizing multiple plasma protein biomarkers. This includes but are not limited to; a large expected dynamic range of the proteins in plasma which can be quite different from protein to protein, obtaining a high degree of multiplexing in the assays used while managing cost vs sample size, and finally restrictions on the sample volume needed^35^. Despite these challenges, we have previously taken a multiplex assay developed with protein measured in NPX^19^ to a validated^20^ version with absolute quantification and these are necessary steps before applying the models here in clinical context. These steps also include retraining the models to fit the new concentration ranges and re-tuning of the cut-off to match the updated model.

The final model presented here for separating benign from malignant cases is based on eight proteins. Several of these have previously been associated with ovarian cancer, either on expression as characterized at gene or protein level (WFDC2, MUC16, KRT19^20^, RBFOX3^23^, CRISP3^36^) or as drug targets (FOLR1^37^), while for instance TCOF1^38^ have been implicated in other cancers but, to the best of our knowledge, not with ovarian cancer. The last protein in the model, LEO1, has been shown^39^ to regulate heterochromatin with downstream importance in maintenance of cellular quiescence. Overall, our results indicate that it would be difficult to carry out a specific pre-selection of what potential biomarkers to characterize either based on previous indications on protein level or based on gene-expression in tumor tissue. Here, only 21% of the potential single valued protein biomarkers showed a strong correlation between tumor gene expression and plasma protein concentrations while the vast majority showed no correlation. One such example is the RBFOX3 protein, which was one of the highest ranked single biomarkers for separating benign from malignant tumors as well as ranked with very high importance in the multivariate models, albeit with weak correlation with its corresponding gene expression (*R* = 0.11). When examining the protein-protein correlation networks, we found RBFOX3 to be part of a larger network of highly correlated plasma proteins out of which 6 other members did show correlation with their corresponding gene expression. This suggests that a large proportion of the potential plasma biomarkers presented here could be downstream effects of the growing tumor or from the tumor microenvironment rather than an underlying driver of the tumor development. This is highly relevant and put restrictions on the interpretation of the proteins in relation to cancer biology and also restricts the possible use of these protein biomarkers as future drug-targets, at least until their role in relation to the disease is understood.

Our study has several limitations. We are limited by the number of samples analyzed in the sense that specific stratification of separate histology in combination with, e.g., stage or other clinical parameters, is not possible given the current material. Although geographically separate, both our cohorts are Swedish and further studies in groups of other ethnicities would have been beneficial to better understand the variation. Neither have we evaluated the models in healthy women, such analyses could give insights into any future application in population screening or early detection in groups with known elevated risk. It is also known that e.g., anthropometrics, life-style variables, or genetics affect circulating plasma protein levels^40–42^ and additional studies are needed to elucidate the contribution of such factors to each of the protein themselves and, to the model-based risk-scores. Lastly, we used the same technology for protein characterizations in the both the discovery and replication cohorts. Although this focused the validation on biology, removing as much technical variance as possible, additional validations of the results using complementary technologies would have further strengthened our results.

In conclusion, our study encompasses the largest plasma proteome study to date in relation to ovarian cancer. Our results include a large number of replicated plasma protein biomarkers for the separation of benign and malignant tumors in symptomatic women, where we also show that only a fraction of these correlate with tumor gene expression. A multivariate model containing eight proteins showed excellent replicated performance in separating benign from malignant tumors regardless of tumor histology and thus could have clinical use as a triaging tool for symptomatic women.

## Supplementary information

Supplementary Material

Description of Additional Supplementary Files

Supplementary Data 1

Supplementary Data 2

Supplementary Data 3

Supplementary Data 4

Supplementary Data 5

Supplementary Data 6

Reporting Summary

## Data Availability

Raw data is located in controlled access data storage at the Swedish Science for Life Laboratories (SciLifeLab) Data Repositories with the following accession numbers: 10.17044/scilifelab.27233412 (protein-data) and 10.17044/scilifelab.28342484 (RNA-data). Results data underlying the figures in this publication can be found in the Supplementary Data 6.

## References

[1] Ferlay, J. et al. Global Cancer Observatory: Cancer Today (version 1.1). Lyon, France: International Agency for Research on Cancer. Available from: https://gco.iarc.who.int/today, accessed [02 March 2024] Cancer Today. https://gco.iarc.fr/today/en (2024).

[2] Froyman, W. et al. Risk of complications in patients with conservatively managed ovarian tumours (IOTA5): a 2-year interim analysis of a multicentre, prospective, cohort study. *Lancet Oncol.* **20**, 448–458 (2019). 30737137 10.1016/S1470-2045(18)30837-4

[3] Menon, U. et al. Ovarian cancer population screening and mortality after long-term follow-up in the UK Collaborative Trial of Ovarian Cancer Screening (UKCTOCS): a randomised controlled trial. *Lancet* **397**, 2182–2193 (2021). 33991479 10.1016/S0140-6736(21)00731-5 PMC8192829

[4] Bast, R. C., Han, C. Y., Lu, Z. & Lu, K. H. Next steps in the early detection of ovarian cancer. *Commun. Med.* **1**, 1–3 (2021). 34676377 10.1038/s43856-021-00037-9 PMC8525879

[5] Chai, C. et al. Single-cell transcriptome analysis of epithelial, immune, and stromal signatures and interactions in human ovarian cancer. *Commun. Biol.* **7**, 1–15 (2024). 38278958 10.1038/s42003-024-05826-1 PMC10817929

[6] de Visser, K. E. & Joyce, J. A. The evolving tumor microenvironment: From cancer initiation to metastatic outgrowth. *Cancer Cell* **41**, 374–403 (2023). 36917948 10.1016/j.ccell.2023.02.016

[7] Menon, U. et al. Sensitivity and specificity of multimodal and ultrasound screening for ovarian cancer, and stage distribution of detected cancers: results of the prevalence screen of the UK Collaborative Trial of Ovarian Cancer Screening (UKCTOCS). *Lancet Oncol.* **10**, 327–340 (2009). 19282241 10.1016/S1470-2045(09)70026-9

[8] Lycke, M., Kristjansdottir, B. & Sundfeldt, K. A multicenter clinical trial validating the performance of HE4, CA125, risk of ovarian malignancy algorithm and risk of malignancy index. *Gynecol. Oncol.* **151**, 159–165 (2018). 30149898 10.1016/j.ygyno.2018.08.025

[9] Jacobs, I. J. et al. Ovarian cancer screening and mortality in the UK Collaborative Trial of Ovarian Cancer Screening (UKCTOCS): a randomised controlled trial. *Lancet* **387**, 945–956 (2016). 26707054 10.1016/S0140-6736(15)01224-6 PMC4779792

[10] Tian, C., Wen, S. Bin, Zhao, C. Y., Yan, X. N. & Du, J. X. Comparative diagnostic accuracy of the IOTA SRR and LR2 scoring systems for discriminating between malignant and Benign Adnexal masses by junior physicians in Chinese patients: a retrospective observational study. *BMC Women's Health* **23**, 585 (2023). 10.1186/s12905-023-02719-z PMC10633950 37940895

[11] Davenport, C. et al. Menopausal status, ultrasound and biomarker tests in combination for the diagnosis of ovarian cancer in symptomatic women. *Cochrane Database Syst. Rev.* **2022**, CD011964 (2022). 10.1002/14651858.CD011964.pub2 PMC9314189 35879201

[12] Kim, S.-Y. & Lee, J. R. Fertility preservation option in young women with ovarian cancer. *Future Oncol.* **12**, 1695 (2016). 27193251 10.2217/fon-2016-0181 PMC5549777

[13] Sölétormos, G. et al. Clinical use of cancer biomarkers in epithelial ovarian cancer: updated guidelines from the european group on tumor markers. *Int. J. Gynecol. Cancer* vol. **26,** 43–51 (2016). 10.1097/IGC.0000000000000586 PMC4679342 26588231

[14] Lycke, M., Ulfenborg, B., Malchau Lauesgaard, J., Kristjansdottir, B. & Sundfeldt, K. Consideration should be given to smoking, endometriosis, renal function (eGFR) and age when interpreting CA125 and HE4 in ovarian tumor diagnostics. *Clin. Chem. Lab. Med.* **59**, 1954–1962 (2021). 34388324 10.1515/cclm-2021-0510

[15] Ding, L. et al. Elevated CA125 levels are associated with adverse clinical outcomes in acute pancreatitis: A propensity score-matched study. *Pancreatology* **20**, 789–794 (2020). 32660761 10.1016/j.pan.2020.06.009

[16] Bulska-Będkowska, W. et al. CA125 as a marker of heart failure in the older women: population-based analysis. *J. Clin. Med.* **8**, 607 (2019). 10.3390/jcm8050607 PMC6572540 31058877

[17] Reilly, G. P. et al. A real-world comparison of the clinical and economic utility of OVA1 and CA125 in assessing ovarian tumor malignancy risk. *J. Comp. Eff. Res.* **12**, 456–472 (2023). 10.57264/cer-2023-0025 PMC10402905 37212790

[18] Coleman, R. L. et al. Validation of a second-generation multivariate index assay for malignancy risk of adnexal masses. *Am. J. Obstet. Gynecol.* **215**, 82.e1–82.e11 (2016). 26970494 10.1016/j.ajog.2016.03.003

[19] Enroth, S. et al. High throughput proteomics identifies a high-accuracy 11 plasma protein biomarker signature for ovarian cancer. *Commun. Biol.* **2**, 221 (2019). 31240259 10.1038/s42003-019-0464-9 PMC6586828

[20] Enroth, S. et al. Data-driven analysis of a validated risk score for ovarian cancer identifies clinically distinct patterns during follow-up and treatment. *Commun. Med.* **2**, 1–13 (2022). 36196264 10.1038/s43856-022-00193-6 PMC9526736

[21] Gyllensten, U. et al. Next generation plasma proteomics identifies high-precision biomarker candidates for ovarian cancer. *Cancers* **14**, 1757 (2022). 35406529 10.3390/cancers14071757 PMC8997113

[22] Álvez, M. B. et al. Next generation pan-cancer blood proteome profiling using proximity extension assay. *Nat. Commun.* **14**, 1–13 (2023). 37463882 10.1038/s41467-023-39765-y PMC10354027

[23] Ivansson, E. et al. Large-scale proteomics reveals precise biomarkers for detection of ovarian cancer in symptomatic women. *Sci. Rep.* **14**, 1–9 (2024). 39068297 10.1038/s41598-024-68249-2 PMC11283551

[24] Region Västra Götaland. Gothia Forum för klinisk forskning: biobank Väst. https://www.gothiaforum.com/web/en.

[25] Glimelius, B. et al. U-CAN: a prospective longitudinal collection of biomaterials and clinical information from adult cancer patients in Sweden. *Acta Oncol.* **57**, 187–194 (2018). 28631533 10.1080/0284186X.2017.1337926

[26] PEA-a high-multiplex immunoassay technology with qPCR or NGS readout. *Mol. Cell Proteom*. **20**, 100168 (202).

[27] Ewels, P. A. et al. The nf-core framework for community-curated bioinformatics pipelines. *Nat. Biotechnol.* **38**, 276–278 (2020). 32055031 10.1038/s41587-020-0439-x

[28] R Core Team. *R: A Language and Environment for Statistical Computing*. (R Foundation for Statistical Computing, 2020).

[29] Network Analysis and Visualization [R package igraph version 2.0.3]. 10.32614/CRAN.PACKAGE.IGRAPH. (2024)

[30] Kuhn, M. Building predictive models in R using the caret package. *J. Stat. Softw.* **28**, 1–26 (2008).27774042

[31] Wang, B. & Zou, H. Another Look at distance-weighted discrimination. *J. R. Stat. Soc. Ser. B Stat. Methodol.* **80**, 177–198 (2018).

[32] CRAN: Package kerndwd. https://cran.r-project.org/web/packages/kerndwd/index.html.

[33] Robin, X. et al. pROC: An open-source package for R and S+ to analyze and compare ROC curves. *BMC Bioinforma.* **12**, 1–8 (2011). 10.1186/1471-2105-12-77 PMC3068975 21414208

[34] Olink Explore HT — Olink®. https://olink.com/products/olink-explore-ht.

[35] Suhre, K., McCarthy, M. I. & Schwenk, J. M. Genetics meets proteomics: perspectives for large population-based studies. *Nat. Rev. Genet.* **22**, 19–37 (2020). 32860016 10.1038/s41576-020-0268-2

[36] Gao, Y. et al. Cross-validation of genes potentially associated with overall survival and drug resistance in ovarian cancer. *Oncol. Rep.* **37**, 3084–3092 (2017). 28350120 10.3892/or.2017.5534

[37] Daigre, J. et al. Preclinical evaluation of novel folate receptor 1-directed CAR T cells for ovarian cancer. *Cancers* **16**, 333 (2024). 10.3390/cancers16020333 PMC10813853 38254822

[38] Yun, H. et al. TCOF1 promotes the colorectal cancer progression by stabilizing *β*-catenin. *Med. Oncol*. **40**, (2023). 10.1007/s12032-023-02218-z 37935810

[39] Oya, E. et al. Leo1 is essential for the dynamic regulation of heterochromatin and gene expression during cellular quiescence. *Epigenet. Chromatin.* **12**, 1–12 (2019). 10.1186/s13072-019-0292-7 PMC6636030 31315658

[40] Enroth, S., Johansson, Å, Enroth, S. B. & Gyllensten, U. Strong effects of genetic and lifestyle factors on biomarker variation and use of personalized cutoffs. *Nat. Commun.* **5**, 4684 (2014). 25147954 10.1038/ncomms5684 PMC4143927

[41] Folkersen, L. et al. Mapping of 79 loci for 83 plasma protein biomarkers in cardiovascular disease. *PLoS Genet.* **13**, 1006706 (2017). 10.1371/journal.pgen.1006706 PMC5393901 28369058

[42] Sun, B. B. et al. Plasma proteomic associations with genetics and health in the UK Biobank. *Nat.* **622**, 329–338 (2023). 10.1038/s41586-023-06592-6 PMC10567551 37794186

