# Supplementary material for: Deep plasma proteomics identifies and validates an eight-protein biomarker panel that separate benign from malignant tumors in ovarian cancer
Source: Commun Med (Lond). 2025 Jun 12;5:230. doi: 10.1038/s43856-025-00945-0 (PMC12162877; doi:10.1038/s43856-025-00945-0)
Supplement: Supplementary file 1 — Supplementary Material [file 43856_2025_945_MOESM1_ESM.pdf]

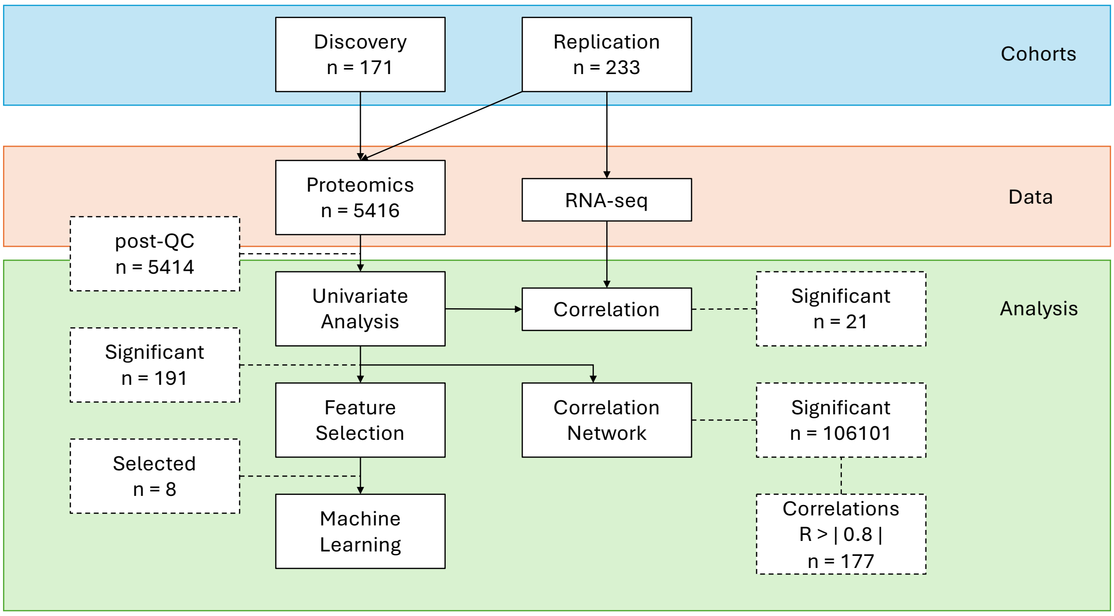

Supplementary Figure 1: Flowchart showing the use of the data and the major analysis steps taken.

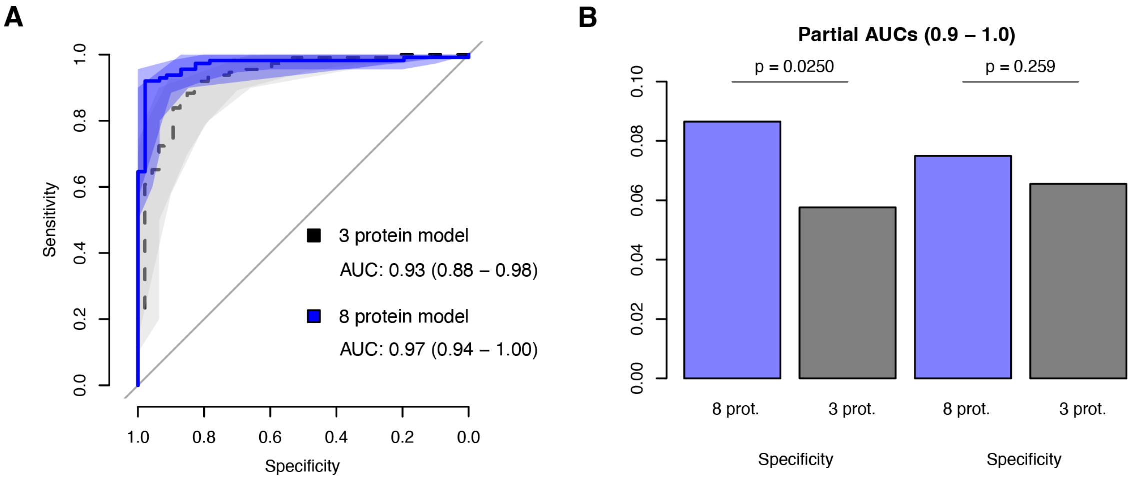

Supplementary Figure 2: Comparison between our previously developed 3-protein model[1] and the current 8-protein model. **A)** ROC-curves for separation of benign and malign tumours. The shaded areas indicate 95% confidence intervals (CI) surrounding the point-estimate ROCs. The 8-protein model is shown with a solid black line with CIs in light blue and the 3-protein model with a dashed black line with Cis in light grey. The AUCs are written out with point-estimates and 95% confidence intervals. **B)** Comparisons of partial AUCs (pAUC) for sensitivity and specificity in the range of 0.9 to 1.0. The 8-protein model is shown in blue

the 3-protein model in grey. Maximum pAUC in the range is 0.1. The p-values above the bars is calculated based the DeLong's method (one-sided).

Supplementary References

1. Ivansson, E. *et al.* Large-scale proteomics reveals precise biomarkers for detection of ovarian cancer in symptomatic women. *Scientific Reports 2024 14:1* **14**, 1–9 (2024).
