# Supplementary material for: Deep plasma proteomics identifies and validates an eight-protein biomarker panel that separate benign from malignant tumors in ovarian cancer
Source: Commun Med (Lond). 2025 Jun 12;5:230. doi: 10.1038/s43856-025-00945-0 (PMC12162877; doi:10.1038/s43856-025-00945-0)
Supplement: Supplementary file 2 — Description of Additional Supplementary Files [file 43856_2025_945_MOESM2_ESM.pdf]

Description of Additional Supplementary Files

File name: Supplementary Data
Description: Underlying data for all main figures.
