# Supplementary material for: Deep plasma proteomics identifies and validates an eight-protein biomarker panel that separate benign from malignant tumors in ovarian cancer
Source: Commun Med (Lond). 2025 Jun 12;5:230. doi: 10.1038/s43856-025-00945-0 (PMC12162877; doi:10.1038/s43856-025-00945-0)
Supplement: Supplementary file 9 — Reporting Summary [file 43856_2025_945_MOESM9_ESM.pdf]

nature portfolio

Corresponding author(s): Stefan Enroth

Last updated by author(s): Feb 4, 2025

Reporting Summary

Nature Portfolio wishes to improve the reproducibility of the work that we publish. This form provides structure for consistency and transparency in reporting. For further information on Nature Portfolio policies, see our Editorial Policies and the Editorial Policy Checklist.

Statistics

For all statistical analyses, confirm that the following items are present in the figure legend, table legend, main text, or Methods section.

n/a	Confirmed	
☐	☒	The exact sample size (*n*) for each experimental group/condition, given as a discrete number and unit of measurement
☐	☒	A statement on whether measurements were taken from distinct samples or whether the same sample was measured repeatedly
☐	☒	The statistical test(s) used AND whether they are one- or two-sided<br>*Only common tests should be described solely by name; describe more complex techniques in the Methods section.*
☐	☒	A description of all covariates tested
☐	☒	A description of any assumptions or corrections, such as tests of normality and adjustment for multiple comparisons
☐	☒	A full description of the statistical parameters including central tendency (e.g. means) or other basic estimates (e.g. regression coefficient) AND variation (e.g. standard deviation) or associated estimates of uncertainty (e.g. confidence intervals)
☐	☒	For null hypothesis testing, the test statistic (e.g. *F*, *t*, *r*) with confidence intervals, effect sizes, degrees of freedom and *P* value noted<br>*Give P values as exact values whenever suitable.*
☒	☐	For Bayesian analysis, information on the choice of priors and Markov chain Monte Carlo settings
☒	☐	For hierarchical and complex designs, identification of the appropriate level for tests and full reporting of outcomes
☒	☐	Estimates of effect sizes (e.g. Cohen's *d*, Pearson's *r*), indicating how they were calculated

Our web collection on statistics for biologists contains articles on many of the points above.

Software and code

Policy information about availability of computer code

Data collection	No software was used for data collection.
Data analysis	R programming language, version 4.2.3 and additional packages within as listed in "Statistics and data analysis"

For manuscripts utilizing custom algorithms or software that are central to the research but not yet described in published literature, software must be made available to editors and reviewers. We strongly encourage code deposition in a community repository (e.g. GitHub). See the Nature Portfolio guidelines for submitting code & software for further information.

Data

Policy information about availability of data

All manuscripts must include a data availability statement. This statement should provide the following information, where applicable:

- Accession codes, unique identifiers, or web links for publicly available datasets
- A description of any restrictions on data availability
- For clinical datasets or third party data, please ensure that the statement adheres to our policy

Raw data for the RNA-sequencing is being deposited in the Federated European Genome Archive (FEGA) but has not yet received an accession number. The proteomics data will be made available in the Swedish Science for Life Laboratory Data Repository (https://figshare.scilifelab.se) under accession number https://doi.org/10.17044/scilifelab.27233412 and through that resource available to researchers on approved request.

Research involving human participants, their data, or biological material

Policy information about studies with human participants or human data. See also policy information about sex, gender (identity/presentation), and sexual orientation and race, ethnicity and racism.

Reporting on sex and gender	This is a study on ovarian cancer and only samples from women have been analysed. No information on gender-identification was available.
Reporting on race, ethnicity, or other socially relevant groupings	Only Swedish women participated. No information on ethnicity was available.
Population characteristics	Diagnose, age, sex and geographic location were the only characteristics available. These are reported in Table 1.
Recruitment	Samples in this study were originally included in research biobanks according to local and national guidelines at participating University hospitals. The samples in this study was collected from the biobanks according to local rules and regulations. Inclusion criteria was suspicion of ovarian cancer followed by surgical diagnosis of either malignant or benign conditions. Exclusion criteria were patients that had received neoadjuvant treatment prior to surgery or if the tumor was pathologically determined to be metastases originating from other tissues.
Ethics oversight	The study was approved by the Regional Ethics Committee in Uppsala (Dnr: 2016/145) and Göteborg (Dnr: 201-15) and informed written consent was obtained from all participants following the guidelines of the Declaration of Helsinki.

Note that full information on the approval of the study protocol must also be provided in the manuscript.

Field-specific reporting

Please select the one below that is the best fit for your research. If you are not sure, read the appropriate sections before making your selection.

☒ Life sciences ☐ Behavioural & social sciences ☐ Ecological, evolutionary & environmental sciences

For a reference copy of the document with all sections, see nature.com/documents/nr-reporting-summary-flat.pdf

Life sciences study design

All studies must disclose on these points even when the disclosure is negative.

Sample size	Plasma samples of women with benign and malignant ovarian tumors were collected from the tumor biobank34 at Biobankvast.se, Western healthcare region, Göteborg, Sweden (N=171) and from the U-CAN collection35 at Uppsala Biobank, Uppsala University, Sweden (N=233). These were the samples available and no prior power calculations were done.
Data exclusions	). In the resulting data-file provided by the analysis platform, each individual protein measurement, assay and sample is labelled depending on passing or failing quality control as provided by the instrument software. Here, a total on 768 individual measurements (corresponding to 0.036%, 768/(404*5416)) did not pass quality control and were removed from further analyses. Two proteins (Apolipoprotein E (APOE) and Fibronektin 1 (FN1)) had detection rates below 95% across all samples and were removed from further analyses.
Replication	Analyses were carried out in two cohorts. One was used strictly as discovery and the second as validation.
Randomization	Samples were analyzed in groups corresponding to clinical diagnose and no randomization was applied. As noted above, training of models were done using one cohort and then externally validated in a second cohort.
Blinding	Blinding is not relevant. This is a biomarker study and e.g. case/control status needs to be known.

Reporting for specific materials, systems and methods

We require information from authors about some types of materials, experimental systems and methods used in many studies. Here, indicate whether each material, system or method listed is relevant to your study. If you are not sure if a list item applies to your research, read the appropriate section before selecting a response.

Materials & experimental systems

n/a	Involved in the study
☐	☒ Antibodies
☒	☐ Eukaryotic cell lines
☒	☐ Palaeontology and archaeology
☒	☐ Animals and other organisms
☒	☐ Clinical data
☒	☐ Dual use research of concern
☒	☐ Plants

Methods

n/a	Involved in the study
☒	☐ ChIP-seq
☒	☐ Flow cytometry
☒	☐ MRI-based neuroimaging

Antibodies

Antibodies used	The underlying assays used here, the Olink Explore uses pairs of antibodies to recognize proteins. There are 5400+ proteins characterized by the assay and individual antibodies used are not disclosed to the end user of the assay.
Validation	This is work carried out by the manufacturer of the assay and not by us.

Plants

Seed stocks	*Report on the source of all seed stocks or other plant material used. If applicable, state the seed stock centre and catalogue number. If plant specimens were collected from the field, describe the collection location, date and sampling procedures.*
Novel plant genotypes	*Describe the methods by which all novel plant genotypes were produced. This includes those generated by transgenic approaches, gene editing, chemical/radiation-based mutagenesis and hybridization. For transgenic lines, describe the transformation method, the number of independent lines analyzed and the generation upon which experiments were performed. For gene-edited lines, describe the editor used, the endogenous sequence targeted for editing, the targeting guide RNA sequence (if applicable) and how the editor was applied.*
Authentication	*Describe any authentication procedures for each seed stock used or novel genotype generated. Describe any experiments used to assess the effect of a mutation and, where applicable, how potential secondary effects (e.g. second site T-DNA insertions, mosiacism, off-target gene editing) were examined.*
